# S100A6, Calumenin and Cytohesin 2 as Biomarkers for Cutaneous Involvement in Systemic Sclerosis Patients: A Case Control Study

**DOI:** 10.3390/jpm11050368

**Published:** 2021-05-02

**Authors:** Paul Balanescu, Eugenia Balanescu, Cristian Baicus, Anca Balanescu

**Affiliations:** 1 Internal Medicine Chair, University of Medicine and Pharmacy Carol Davila, 020021 Bucharest, Romania; cristian.baicus@umfcd.ro; 2 Clinical Immunology Laboratory CDPC, Colentina Clinical Hospital, 020125 Bucharest, Romania; dzum_dzum@yahoo.com; 3 Clinical Research Unit RECIF (Reseau d’Epidemiologie Clinique International Francophone), 020125 Bucharest, Romania; 4 Pediatrics Chair, University of Medicine and Pharmacy Carol Davila, 020021 Bucharest, Romania; anca.balanescu@umfcd.ro

**Keywords:** systemic sclerosis, biomarker, calumenin, S100A6, cytohesin 2

## Abstract

Background: Systemic sclerosis (Ssc) is an autoimmune disease with incomplete known physiopathology. There is a high number of candidate proteomic biomarkers for Ssc that have not yet been confirmed on independent Ssc cohorts. The aim of the study was to confirm circulating S100A6, calumenin, and cytohesin 2 as biomarkers for Ssc. Methods: 53 Ssc patients and 26 age- and gender-matched controls were included. Serum S100A6, calumenin, and cytohesin 2 were evaluated with commercial ELISA kits. Associations between serum expression and clinical Ssc characteristics were evaluated. Results: Serum calumenin, S100A6, and cytohesin 2 were higher in Ssc patients compared to controls. Calumenin associated with extensive cutaneous fibrosis, frequency of Raynaud phenomenon, and low complement level, and had a tendency to be higher in Ssc patients with pulmonary fibrosis. S100A6 correlated with the number of active digital ulcers. Serum cytohesin 2 levels were higher in patients with teleangiectasia and associated with pulmonary artery pressure. Conclusions: Serum calumenin, S100A6, and cytohesin 2 were confirmed as biomarkers on an independent group of Ssc patients. Calumenin had the best predictive capacity for cutaneous Ssc manifestations. Future studies are needed to evaluate the prognostic value of these biomarkers and evaluate them as possible therapeutic targets.

## 1. Introduction

Systemic sclerosis (Ssc) is a systemic autoimmune disease with incomplete known physiopathology. Its hallmark is represented by collagen deposition in various tissues that are accompanied by vascular injury [1]. As the clinical course of the disease can be severe in later Ssc stages, predictive biomarkers for severity and Ssc disease activity are necessary for better management of these patients [2]. Proteomic biomarkers are relatively easy to determine in the clinical laboratory setting using usual medical laboratory equipment. As such, there is an increased interest in research for proteomic biomarkers in Ssc patients. Previous mass spectrometry study pinpointed a relatively large number of candidate biomarkers for Ssc patients [3,4]. Nevertheless, the number of confirmed biomarkers on independent Ssc cohorts using specific assays (based on specific antibody-antigen reaction) is small. As such, studies are needed in order to confirm and evaluate the predictive value of candidate biomarkers on independent Ssc patient groups.

The study aimed to confirm circulating S100A6, calumenin, and cytohesin 2 as biomarkers for Ssc. These molecules were previously identified from various biological Ssc samples as candidate biomarkers. To the best of our knowledge, this is the first confirmatory study for these molecules as circulatory biomarkers in Ssc patients.

## 2. Materials and Methods

### 2.1. Patient Evaluation

Fifty-three consecutive Ssc patients that attended Colentina Clinical Hospital between January 2013 and January 2018 were included in the analysis (4 males and 49 females, median age 56 years). Patients were clinically evaluated and their cutaneous and visceral disease involvement was assessed.

Interstitial lung disease (ILD) was determined using X-ray imaging and high resolution CT. Flow spirometry and diffusing capacity for carbon monoxide-DLCO was determined in these patients (QUARK PFT station, Cosmed, Rome, Italy).

Pulmonary hypertension was indirectly determined by transthoracic echocardiography (Hitachi Aloka SSD 4000 ultrasound machine, Hitachi, Japan). Possible pulmonary hypertension was considered when the patients had at least 35 mm Hg at rest in pulmonary artery [5]. Cardiac involvement was determined using cardiac ultrasonography and EKG (Cardiocontrol, MDIMedical, Belfast, UK).

Frequency of Raynaud was interpreted as daily or less frequent. Presence of telangiectasias, ischemic digital ulcers, and calcinosis were recorded as previously defined in the literature [6]. Severity of skin involvement was evaluated using modified Rodnan skin score [7].

Disease activity score was evaluated with EUSTAR criteria [8]. Gastrointestinal involvement was evaluated using upper gastrointestinal endoscopy and radiographic examinations including barium swallow for esophageal motility. Inflammatory markers were also recorded in Ssc patients (erythrocyte sedimentation rate (ESR) and serum C reactive protein (CRP) concentration). Patient medication at the time of enrollment was also recorded.

Twenty-six age- and gender-matched controls were recruited in this study (2 males, 24 females, median age 51.5 years) from patients referred to Colentina Clinical Hospital. Controls had no previous record of Raynaud’s phenomenon and no active inflammation.

Patients and controls signed the informed consent. The study was conducted according to the Declaration of Helsinki and was approved by the local ethics committee (decision No. 10/11 September 2020).

### 2.2. Sample Evaluation

Serum was separated and stored in an ultrafreezer at −80 °C. Indirect immunofluorescence was used for antinuclear antibodies analysis (ANA). Commercial enzyme linked immunosorbent assay (ELISA) kits were used to determine anti-Scl70 and anti-centromere antibodies (Euro Diagnostica, Malmo, Sweden).

#### 2.2.1. S100A6 Assay

Serum S100A6 levels were determined using commercial sandwich ELISA kit (ELH-S-100A6-1 Raybiotech, Peachtree Corners, GA, USA) according to the manufacturer’s instructions. The intra-assay and inter-assay coefficient of variation were 10% and 12%. The minimum detectable concentration of S100A6 was 0.06 ng/mL.

#### 2.2.2. Calumenin Assay

Serum calumenin was determined using commercial sandwich ELISA kit (ELH-CALU-1 Raybiotech, Peachtree Corners, GA, USA) according to the manufacturer’s instructions. The intra-assay and inter-assay coefficient of variation were 10% and 12%. The minimum detectable concentration of calumenin was 0.55 ng/mL.

#### 2.2.3. Cytohesin 2 Assay

Serum cytohesin 2 levels were assayed using commercial competitive ELISA kit (ABIN857811, MyBiosource, San Diego, CA, USA). The intra-assay and inter-assay coefficient of variation were 10% and 10%. The minimum detectable concentration of cytohesin 2 was 0.1 ng/mL.

All samples were analyzed in duplicate, with optical densities evaluated at 450 nm using a microplate reader (Dynex, Buštěhrad, Czech Republic). In order to evaluate the standard curve and estimate the concentration of the samples, a four-parameter logistic regression curve was used (Aladyn software, Prague, Czech Republic). Results were presented as nanograms per milliliter (ng/mL).

### 2.3. Statistical Analysis

Continuous variables were presented as median and interquartile range (IQR) value if a non-normal distribution was found. Normality of distribution was tested with Kolmogorov-Smirnov tests. Fisher’s exact tests were used to evaluate association between categorical variables. Differences between continuous variables were evaluated with Mann-Whitney U test. Spearman rho coefficient was used in order to assess correlations in continuous variables. A threshold for *p*-value ≤ 0.05 was considered significant. Statistical Software for Social Sciences (SPSS), Version 16 for Windows (Chicago, IL, USA) was used.

## 3. Results

Descriptive data of patients included in Ssc group and healthy controls are presented in Table 1.

### 3.1. Serum Calumenin in Ssc Patients

**Figure 1:**
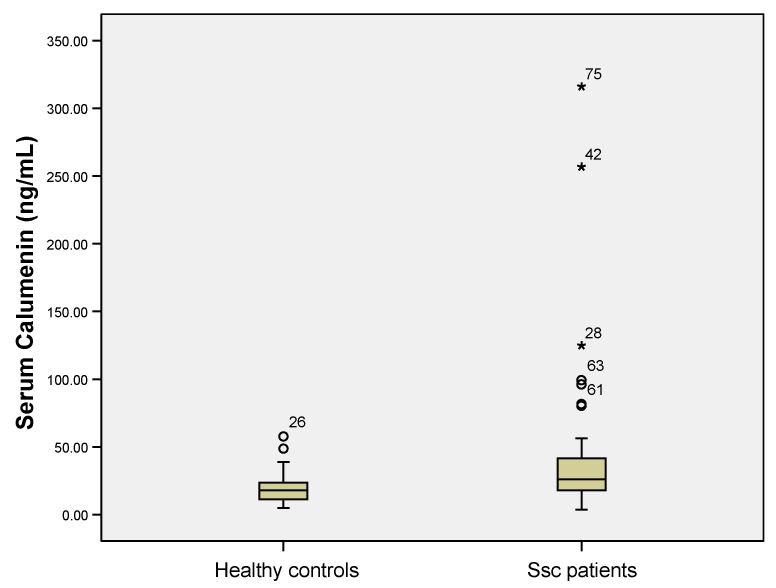
Serum calumenin levels were higher in Ssc (systemic sclerosis) patients compared to healthy controls (*p* = 0.002, Mann Whitney U test). The circles, stars and numbers in figure represent the patient ID in the database that are considered outliers by the statistical software used.

Serum calumenin levels were higher in Ssc patients compared to controls (median 26.14 ng/mL (interquartile range—IQR 27.22) versus median 17.97 ng/mL (13.82), Mann Whitney U test, *p* = 0.002, Figure 1). Ssc patients with a modified Rodnan score ≥ 14 (thus with diffuse cutaneous fibrosis) had higher circulatory calumenin levels. Median calumenin levels in Ssc patients with diffuse cutaneous fibrosis were 30.55 ng/mL (30.12) versus 20.08 ng/mL (16.57), Mann Whitney U test, *p* = 0.015

**Figure 2:**
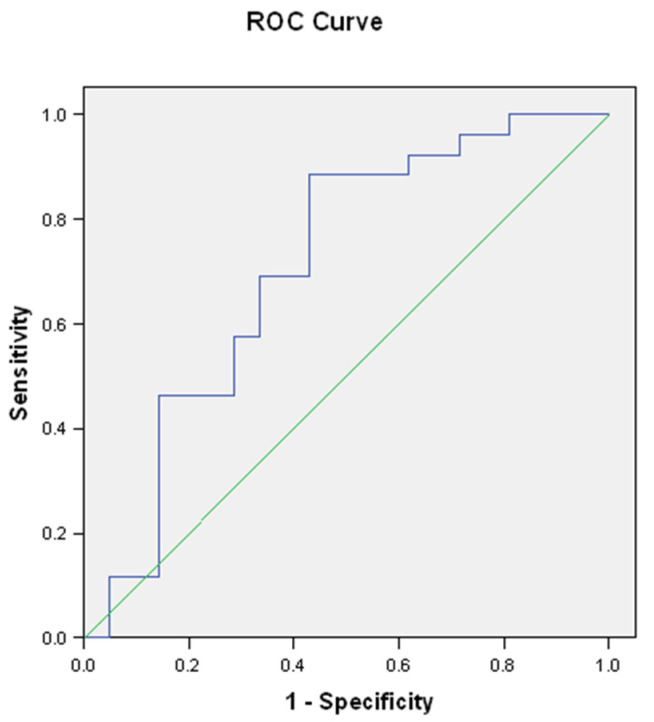
ROC curve for calumenin as diagnostic test for diffuse cutaneous fibrosis. AUROC = 0.70, 95% confidence interval 0.56–0.86.

When used as a diagnostic test for diffuse cutaneous fibrosis, calumenin had a relatively good diagnostic capacity AUROC = 0.70 (95% confidence interval (0.56–0.86)), Figure 2.

In addition, patients with daily or higher frequency of Raynaud phenomenon had higher calumenin levels (30.36 ng/mL (35.11) versus 17.84 ng/mL (12.24), Mann Whitney U test, *p* = 0.006).

Patients with low C3 or C4 levels also had significantly higher calumenin levels (median 54.34 ng/mL (116.04) versus 23.71 ng/mL (15.19), Mann Whitney U test, *p* = 0.03). Circulating calumenin levels were positively correlated with modified Rodnan scores (r = 0.40, *p* = 0.004) with CRP levels (r = 0.343, *p* = 0.02) and with ESR (r = 0.372, *p* = 0.01).

Interestingly, patients with pulmonary fibrosis had a tendency for higher circulatory calumenin levels (30.22 ng/mL (34.62) versus 21.5 ng/mL (12.70) *p* = 0.087, Mann Whitney U test). Nevertheless, no significant differences between serum calumenin levels in diffuse versus localized Ssc patients were found.

### 3.2. Serum S100A6 in Ssc Patients

**Figure 3:**
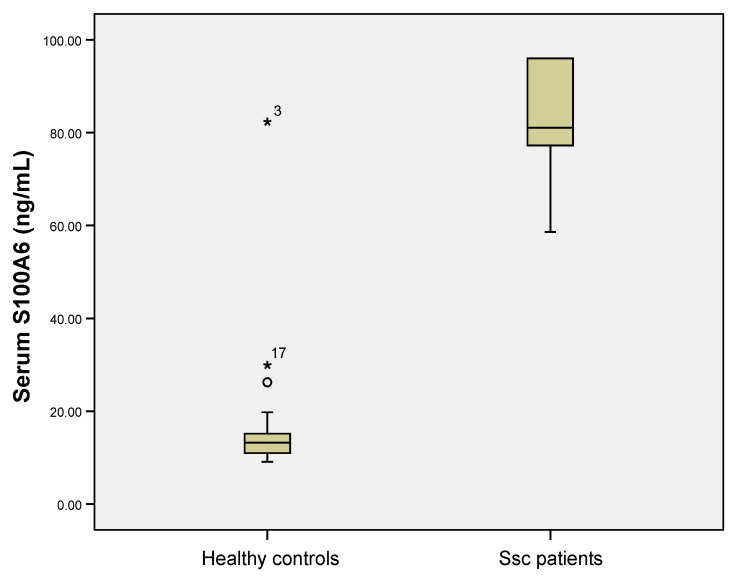
Serum S100A6 levels were higher in Ssc (systemic sclerosis) patients compared to healthy controls (*p* < 0.001, Mann Whitney U test). The circle, stars and numbers in figure represent the patient ID in the database that are considered outliers by the statistical software used.

Serum S100A6 levels were statistically significant higher in the Ssc group compared to controls (median 81.06 ng/mL (18.80) versus median 13.24 ng/mL (4.85), Mann Whitney U test, *p* < 0.001, Figure 3).

Higher circulatory S100A6 levels were associated with digital ulcer presence (median 91.83 ng/mL in Ssc patients with digital ulcers (17.79) versus median 79.28 ng/mL in Ssc patients without digital ulcers (10.77), Mann Whitney U test, *p* = 0.002). Also, S100A6 levels were positively correlated with the number of active digital ulcers (r = 0.33, *p* = 0.02)

There were no significant differences between serum S100A6 levels in diffuse versus localized Ssc patients, nor with current Ssc medication.

### 3.3. Serum Cytohesin 2 in Ssc Patients

**Figure 4:**
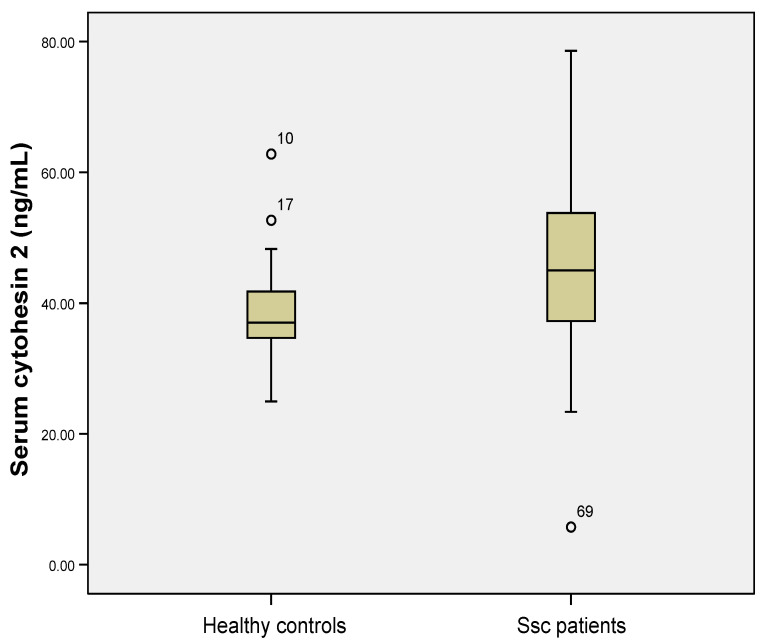
Serum cytohesin 2 levels were higher in Ssc (systemic sclerosis) patients compared to healthy controls (*p* < 0.001, Mann Whitney U test). The circle and numbers in figure represent the patient ID in the database that are considered outliers by the statistical software used.

Serum cytohesin 2 levels were statistically significant higher in Ssc patients compared to controls (median 45.02 ng/mL (17.07) versus median 37.02 ng/mL (7.15), Mann Whitney U test, *p* = 0.003, Figure 4).

Higher serum cytohesin 2 levels were observed in patients with telangiectasia presence (median 45.11 ng/mL (19.53) versus 40.46 ng/mL (11.68), Mann Whitney U test, *p* = 0.025).

Palpable tendon friction rubs were associated with lower circulating levels of cytohesin 2 (median 38.63 ng/mL (19.64) versus 46.06 (16.26), Mann Whitney U test, *p* = 0.021). Serum cytohesin 2 levels were positively correlated with inflammatory markers (ESR r = 0.324, *p* = 0.024, CRP r = 0.486, *p* = 0.001). There was a tendency for higher cytohesin 2 levels in patients with active disease, but without statistical significance (*p* = 0.22).

A cut-off value (53.97 ng/mL) defined as mean + two standard deviations of cytohesin 2 value of healthy controls was used to divide Ssc patients into two groups: high cytohesin 2 level and normal cytohesin 2 level. A total number of 13 Ssc patients (24.5%) had high cytohesin 2 levels. These patients had statistically significant higher PAP (pulmonary artery pressure) compared with patients with normal cytohesin 2 levels (median 28 mm Hg (20) versus median 25 mm Hg (2), Mann Whitney U test, *p* = 0.03). There were no significant differences between serum cytohesin 2 levels in diffuse versus localized Ssc patients, nor with current Ssc medication.

### 3.4. Correlations between Calumenin, S100A6 and Cytohesin 2

Calumenin levels were positively correlated with S100A6 levels (r = 0.400, *p* < 0.001), while a positive correlation was observed between S100A6 levels and cytohesin 2 levels (r = 0.262, *p* = 0.02).

## 4. Discussion

The results of the current study confirmed circulatory calumenin, S100A6 and cytohesin 2 as biomarkers for SSc patients. To the best of our knowledge, this is the first study that has evaluated circulatory levels of calumenin, S100A6, and cytohesin 2 on an independent cohort of Ssc patients.

Calumenin is a protein that binds calcium being localized in the endoplasmic reticulum. It belongs to a CREC chaperone alongside with Reticulocalbin 1, Cab45, and ERC-55, being involved in protein folding and the secretory pathway. Calumenin was described to be secreted in the surrounding medium [9]. It has been widely evaluated in various cancers by influencing tumorigenesis and therapeutic response [10]. Increased expression of calumenin was associated with poorer outcome in gliomas [11]. As such, this molecule could play a prognostic role for survival and for the therapeutic response in cancer. Secreted calumenin proved to have autocrine and paracrine effects, being linked to thrombosis and wound healing by modifying the fibroblast phenotype. [12]. Previous data suggest that calumenin is also secreted by activated thrombocytes and is localized in atherosclerotic lesion [13]. Having such an influence upon wound healing via fibroblast activity modulation calumenin could be an interesting and promising biomarker for Ssc patients as it has been previously isolated from biological samples from Ssc patients using mass spectrometry [14]. In this study, calumenin was confirmed as a circulatory biomarker and was associated with diffuse cutaneous fibrosis, inflammation, and low complement level. These data suggests that patients with higher inflammatory activity (that is continuously sustained by complement consumption) associate higher circulatory calumenin levels. However, no association was found between Ssc disease activity score and serum levels of calumenin and between patients with diffuse and localized Ssc. There was also a tendency for higher calumenin levels in patients with pulmonary fibrosis. Although the results did not reach statistical significance, this was most probably due to the relatively small sample size. The regulatory function of calumenin in tissues that are affected by the processes of cytoskeleton rearrangement points out calumenin as a candidate prognostic biomarker in Ssc patients, like has been previously suggested for another member of the CREC family (reticulocalbin 1) [15]. Future prospective studies including early Ssc patients should evaluate the prognostic capacity of circulatory calumenin in Ssc patients.

S100A6 (calcyclin) was extensively evaluated as a biomarker for digestive cancers. Serum S100A6 has a relatively good diagnostic capacity for cholangiocarcinoma [16]. Significant expression of S100A6 was observed also in placenta of women diagnosed with preeclampsia, and its expression is increased in oxidative stress response. [17]. S100A6 was much higher in Ssc patients compared to healthy controls. There were no associations with clinical Ssc features except for higher S100A6 levels in patients with active digital ulcers. Also, serum S100A6 positively correlated with the number of active digital ulcers. These data suggest that S100A6 could be a biomarker associated with more aggressive Ssc pattern. Another striking observation is the fact that S100A6 expression was a few folds higher in Ssc patients compared to controls. S100A6 is linked to the RAGE (Receptor for Advanced Glycation Endproducts) family by interacting with these molecules and acting as cytokines. It seems that S100A6 binding activates the NF-kB pathway and leads to apoptosis [18]. RAGE are known to be associated with various inflammatory cutaneous diseases (both autoimmune and infectious) [19]. RAGE pathway was reported to be involved in Ssc ulcerations development [20]. As a promotor for RAGE pathway, S100A6 could initiate and sustain the inflammatory process that is associated with digital ulceration. Also, very high levels of S100A6 were found in Ssc patients, which points out the fact that this specific molecule could be a potential future therapeutic target at least for prevention of digital ulcer development/aggravation. This hypothesis needs further testing in specific clinical settings on independent Ssc cohorts.

Cytohesin 2 is a guanine-nucleotide exchange factor (GEF) for ARF6 (ADP-ribosylation factor 6). It was previously showed that cytohesin 2 is localized on the plasma membrane and endosome [21]. It is involved in a multitude of signaling and trafficking pathways as it plays a major role in granule secretion in thrombocytes, chromaffin, and pancreatic β-cells [22]. Cytohesin 2 is an activator of both EGF and IGF-1 signaling pathways by direct interaction with the cytoplasmic domains of the activated EGF-R. In vitro studies on colorectal cells pointed out that cytohesin 2 blockade associated with a decreased cell proliferation, migration, and invasion of tumor cells, indicating cytohesin 2 as a potential therapeutic target in colorectal cancer [23]. A recent report also showed that inhibition of cytohesin 2 prevented the morphological changes in vascular smooth muscle cells due to resistin. Cytohesin 2 blockade modified the secretome of the vascular smooth muscle by decreasing matrix metalloproteinase expression [24].

IGF-1 and vascular smooth muscle cells are important pathogenic factors in Ssc, as they also seem to be involved in the progression from preclinical stages of Ssc to early stages [25]. Serum cytohesin 2 levels were increased in Ssc patients, but the prognostic value of these observed changes is not clear. Associations with some cutaneous features of Ssc were pointed out in this study (telangiectasia) alongside with positive correlations with inflammatory markers (especially CRP). Higher cytohesin 2 levels were associated with higher PAP but otherwise no associations with pulmonary Ssc involvement were found, although previous proteomic studies identified cytohesin 2 as a proteomic biomarker from BALF and suggested it as a candidate biomarker for pulmonary involvement in Ssc [14]. This is probably due to the fact the significant changes of cytohesin 2 levels are local rather than systemic. Confirmation studies for cytohesin 2, as a biomarker for IPF from BALF, are needed.

As previous findings pointed out that cytohesin 2 blockade is associated with a more quiescent cellular phenotype, it suggests that cytohesin 2 overexpression could also have a pathogenic effect in Ssc patients. Cytohesin 2 is localized at the crossroad of multiple pathogenic pathways in Ssc patients, and future in vitro studies on fibroblasts derived from Ssc samples are needed in order to evaluate cytohesin 2 blockade effects in Ssc derived cells and define cytohesin 2 as a potential Ssc therapeutic target.

The present study has important limitations. Firstly, the sample size is relatively small, especially for associations between serum levels of the biomarkers and all clinical characteristics of Ssc patients. This was due to the fact that the study was unicentric and Ssc is a rare disease. Secondly, this was only a confirmatory study of the proposed candidate biomarkers, without follow-up and dynamic evaluation of patients in order to validate them as biomarkers in Ssc patients. Thirdly, nailfold capillaroscopy data were not collected for these patients. As such, associations between nailfold capillaroscopy features and biomarkers were not evaluated. Future prospective validation studies on larger Ssc cohorts that also include nailfold capillaroscopy analysis are needed. Prospective studies evaluating the prognostic value of these biomarkers that include Ssc patients with early disease are of great interest.

## 5. Conclusions

In conclusion, this study confirmed circulatory cytohesin 2, calumenin and S100A6 as biomarkers in Ssc patients and found associations between the serum levels of these biomarkers and clinical characteristics, especially with severe cutaneous involvement (necrosis and diffuse sclerosis). Among all these biomarkers, calumenin had the best predictive capacity for cutaneous diffuse manifestation, while S100A6 was associated with digital ulcers. Independent confirmation of candidate biomarkers is the first essential step, and once the biomarkers are validated, it will eventually lead to a personalized approach of Ssc patients.

## Figures and Tables

**Table 1:** Characteristics of included Ssc patients and healthy controls.

Ssc Patient Characteristics	All Ssc Patients (*n* = 53)	Healthy Controls (*n* = 26)	Diffuse Ssc (*n* = 33)	Localized Ssc (*n* = 20)	*p*-Value (between Diffuse SSc and Localized SSc)
Age (median (IQR), years)	56 (15.75)	51.5 (12.50)	54 (14.25)	60.5 (20.25)	0.71 (NS)
Male/Female (n° of patients)	4/49	2/24	3/30	1/19	1 (NS)
Ssc disease duration (median (IQR), months)	84 (94.50)	N/A	102 (95)	77 (96)	0.22 (NS)
Interstitial lung disease (n° of patients)	28 (52.8%)	N/A	21 (63.6%)	7 (35%)	0.04
% DLCO (median (IQR)	78 (22)	N/A	77 (24)	85.5 (15.5)	0.10 (NS)
Pulmonary hypertension (n° of patients)	7 (13.2%)	N/A	3 (9.1%)	4 (20%)	0.26 (NS)
% predicted FVC (median (IQR))	97 (19.5)	N/A	98 (18)	96 (26.75)	0.83 (NS)
Presence of Raynaud phenomenon (n° of patients)	53 (100%)	N/A	33 (100%)	20 (100%)	1 (NS)
Digital ulcers (n° of patients)	12 (22.6%)	N/A	12 (36.4%)	0 (0%)	0.03
No of digital ulcers (median (IQR))	0 (2)	N/A	0 (2)	0 (0)	0.001
Presence of telangiectasia (no of patients)	27 (51%)	N/A	16 (48.5%)	11 (55%)	0.64 (NS)
Palpable friction rubs (n° of patients)	18 (34%)	N/A	14 (42.4%)	4 (20%)	0.10
PAP (median (IQR) mm Hg)	25 (4)	N/A	25 (4)	25 (8)	0.72 (NS)
ANA (n° of patients)	53 (100%)	N/A	33 (100%)	20 (100%)	1 (NS)
Presence of anti-centromere antibodies (n° of patients)	20 (37.7%)	N/A	0 (0%)	20 (100%)	< 0.001
Presence of anti Scl-70 antibodies (n° of positive patients)	23 (43.4%)	N/A	23 (70%)	0 (0%)	0.002
CRP (median (IQR) mg/L)	3.53 (7.28)	N/A	3.98 (11.24)	3.34 (6.44)	0.45 (NS)
ESR (median (IQR) mm/1 h)	16 (16.5)	N/A	16.5 (15.5)	14 (18.50)	0.38 (NS)
Hypocomplementemia (n° of patients)	6 (11.3%)	N/A	4 (12.1%)	2 (10%)	0.81 (NS)
EUSTAR score (median (IQR))	3 (2.50)	N/A	3.50 (2.75)	1.75 (2)	0.001
Steroid therapy (n° of patients)	30 (56.6%)	N/A	18 (54.4%)	12 (60%)	0.69 (NS)
Calcium blockers (n° of patients)	16 (30.2%)	N/A	10 (30.3%)	6 (30%)	0.98 (NS)
ACE inhibitors (n° of patients)	11 (20.7%)	N/A	8 (24.3%)	3 (15%)	0.42 (NS)

NS—not significant, PAP—pulmonary artery pressure, DLCO—diffuse lung capacity for carbon monoxide, ANA—antinuclear antibody, ESR—erythrocyte sedimentation rate; CRP—C-reactive protein; ACE—angiotensin-converting enzyme. n°—number; N/A—not applicable.

## Data Availability

The data presented in this study are available upon reasonable request from the corresponding author.
